# Driving and public transit barriers to dental care in the United States

**DOI:** 10.1016/j.ssmph.2026.101907

**Published:** 2026-03-10

**Authors:** Md Shahinoor Rahman, Ichiro Kawachi, Benjamin D. Sommers, Renuka Tipirneni, Jeffrey C. Blossom, Hawazin W. Elani

**Affiliations:** a Department of Oral Health Policy and Epidemiology at the Harvard School of Dental Medicine, Boston, Massachusetts, USA; b School of Public Health at LSU Health Sciences Center New Orleans, Louisiana, USA; c Department of Social and Behavioral Sciences at the Harvard T. H. Chan School of Public Health, Boston, Massachusetts, USA; d Department of Health Policy and Management at the Harvard T. H. Chan School of Public Health, Boston, Massachusetts, USA; e Divisions of General Medicine and Hospital Medicine, University of Michigan Department of Internal Medicine, Ann Arbor, Michigan, USA; f Center for Geographic Analysis, Harvard University, Cambridge, Massachusetts, USA; g Department of Medicine, Brigham and Women’s Hospital, Harvard Medical School, Boston, Massachusetts, USA; h Institute for Healthcare Policy and Innovation, University of Michigan, Ann Arbor, USA; i Department of Health Management and Policy, School of Public Health, University of Michigan, Ann Arbor, USA; j Division of Hospital Medicine, Department of Internal Medicine, University of Michigan, Ann Arbor, USA

**Keywords:** Travel time, Transit equity, Dental clinic access, Travel time disparities, Urbanicity

## Abstract

Despite the increasing number of dentists in the United States, the uneven distribution of dental clinics and inequities in public transportation contribute to ongoing disparities in access to dental care. This study examined travel times by car and public transit to the nearest dental clinic across the United States. We used data on 104,695 clinics from the IQIVIA practitioners' database. Drive and transit times to the nearest dental clinic were calculated at the block group level. Longer travel-time hotspots were defined as areas where the Getis-Ord Gi∗ z-score was ≥1.96, corresponding to statistical significance at the 95% confidence level. Approximately 49.3 million (19.1%) of US adults lack public transit access to dental care. Mean public transit times were 13.0, 18.5, and 34.3 min in urban, suburban, and rural areas, respectively, while mean driving times were 2.8, 3.4, and 10.5 min. Block groups experiencing extreme social deprivation were 1.7 times more likely (95% CI: 1.5–2.0) to be in drive-time hotspots, and 3.7 times more likely (95% CI: 3.2–4.3) compared with those with the least deprivation. Suburban and rural block groups were 1.4 times (95% CI: 1.3–1.6) and 2.4 times (95% CI: 2.2–2.7), respectively, more likely to be in public-transit-time hotspots than urban block groups. These findings demonstrate significant transportation barriers to accessing dental care across different regions and demographic groups, highlighting disparities in geographic accessibility.

## Highlights

• The uneven distribution of clinics results in disparities in access to dental care.
• 49.3 million (19.1%) of US adults lack public transit access to dental care.
• The mean transit times were 13.0, 18.5, and 34.3 min in urban, suburban, and rural areas.
• Block groups with extreme social deprivation are likely to be in travel time hotspots.
• There are disparities in travel time to the nearest dental clinic in the US.

## 1 Introduction

Transportation barriers significantly limit access to healthcare services, including dental care (Castillo et al., 2023; Ghanbarzadegan et al., 2021; Syed et al., 2013). Despite an increase in the number of dentists (Munson & Vujicic, 2021), the uneven distribution of dental clinics exacerbates transportation barriers, particularly in rural regions (Rahman et al., 2024). For example, in Nevada, 11% of residents have been unable to visit a dentist due to clinic locations, with rural areas being disproportionately affected (Chen, 2021). Similarly, in Washington, D.C., Census tracts with predominantly Black populations are significantly farther from dental clinics, contributing to geographic and racial disparities in access to dental services (Liu et al., 2022).

The financial burden of transportation and long commutes further complicates access to dental services. Individuals with limited resources or without personal vehicles often have to prioritize proximity over quality or cost when selecting a clinic (Arcury et al., 2005; Cho et al., 2022; Comber et al., 2011; Victoor et al., 2014). Public transit, an important enabler for many without personal vehicles, frequently fails to adequately serve marginalized communities, including older adults, individuals with disabilities, and low-income individuals (Chattopadhyay, 2008; Heaps et al., 2021). Black and Hispanic populations, as well as low-income groups, are more likely to use public transportation for accessing healthcare services, making them more vulnerable to the negative consequences of inadequate transit systems, including delayed dental care, missed appointments, and reduced access to preventive care (Bhoopathi et al., 2021; Guo et al., 2022; Labban et al., 2023; McKernan et al., 2016; Smith et al., 2022). For example, 11% of Medicaid-insured adults in Iowa reported unmet dental care needs due to transportation barriers (Marchan et al., 2022). In this study, we consider public transit accessibility not only as a measure of existing travel routes but also as an indicator of potential access, reflecting how transportation infrastructure can enable or constrain mobility for populations dependent on transit.

In 2022, 21% of U.S. adults without access to a vehicle or adequate public transit missed medical care due to transportation barriers (Smith et al., 2023). One policy lever for addressing this concern is Medicaid's coverage of non-emergency medical transportation (NEMT) services (GAO, 2016). State Medicaid programs are mandated to provide NEMT to beneficiaries, although service delivery and eligibility criteria can vary among states (Musumeci & Rudowitz, 2016). Medicare offers limited coverage for NEMT, with only specific medically necessary conditions covered under traditional Medicare. However, in 2024, 36% of Medicare Advantage plans included NEMT benefits (Freed et al., 2023), indicating a growing recognition of these needs.

Transportation is a fundamental but understudied dimension of oral health equity. Driving and public transit each represent distinct mechanisms through which structural and spatial inequalities affect access to dental care. Driving time reflects the geographic availability of clinics and the built environment that supports personal vehicle use, whereas public transit time captures mobility constraints faced by individuals without private vehicles. These transportation factors intersect with neighborhood context, racial and ethnic segregation, and socioeconomic deprivation, that are core social determinants of health that shape both where dental clinics are located and who can realistically reach them.

Despite the importance of transportation in accessing dental care, there remains a limited understanding of travel times required to access dental care across the U.S., especially via public transportation. Moreover, despite common perceptions of affluence in suburban areas, nearly 40% of the uninsured population resides in these areas due to millions of people living in poverty, which limits access to healthcare (Schnake-Mahl & Sommers, 2017). Thus, understanding travel time barriers to accessing dental care across various dimensions, such as neighborhood types (e.g. urban, suburban, and rural areas), racial/ethnic segregation, and socioeconomic deprivation, is crucial for addressing disparities in access to care. Because transit networks are often incomplete or absent in many regions, particularly in rural and suburban areas, examining where public transit access is unavailable is itself informative of structural inequities in transportation and healthcare access. This study aims to examine disparities in travel times by car and public transit to the nearest dental clinic, with the goal of assessing the role of transportation in accessing dental care.

## 2 Methods

### 2.1 Dentist database

We used the IQIVIA national dental provider database, updated in October 2023 (Rahman et al., 2024). This comprehensive database includes a total of 205,762 active dentists and provides detailed information on dental practice locations across the U.S (IQIVIA, 2023). Because our analysis focused on adult access to routine dental care, we excluded dentists who exclusively practice in specialty areas (n = 9006), including oral and maxillofacial surgery, orthodontics, pediatric dentistry, endodontics, periodontics, and prosthodontics. We then aggregated individual dentists by clinic location, resulting in a total of 104,695 unique clinics, and all subsequent analyses were conducted at the clinic level (Supplementary Methods).

### 2.2 Travel time outcomes: driving time and public transit time

We calculated travel times from block group population mean centers to the nearest dental clinic using two modes of transportation: driving and public transit (buses, metro, and train). Driving times were calculated using the Environmental Systems Research Institute (ESRI) ArcGIS StreetMap Premium OD Cost Matrix, which provides reliable routing based on historical traffic data in ArcGIS Pro 3.2.2 (Supplementary Methods) (ESRI, n.d.). We used the travel time impedance attribute as the measure of drive time from block groups to the nearest dental clinic.

Because the ESRI suite does not include a default public-transit network and nationwide General Transit Feed Specification (GTFS) data are incomplete and inconsistently updated, we relied on the Google Maps Distance Matrix API to calculate public-transit times. Google integrates GTFS and other data sources from transit agencies, offering a more comprehensive and current representation of U.S. transit systems than could be achieved by constructing a custom GTFS network within ESRI.

Public transit times were computed using the Google Maps Distance Matrix API (Supplementary Methods) (Google, 2024a). We specified a Tuesday at 10:00 a.m. local time as a standardized, non–rush-hour period to minimize regional variability in congestion and ensure comparability of travel times across all block groups nationally (Chávez & Manaugh, 2025; Lindner et al., 2024). Public transit was considered unavailable when the Google Maps API returned “Zero_Results” for the trip between the population mean center of a block group (origin) and its nearest dental clinic (destination). This return code indicates that no valid public transit route exists between the two locations (Google, 2024b). Block groups with “Zero_Results” were coded as lacking public transit access, and the adult population within these block groups was summed to estimate the total number of U.S. adults without public transit access to a dental clinic.

### 2.3 Socioeconomic data

We merged multiple socio-demographic datasets from the 2022 five-year American Community Survey (ACS), including the number of adult population, racial and ethnic composition, and the percentage of uninsured adult population for each block group, with the U.S. Census Bureau's topologically Integrated Geographic Encoding and Referencing (TIGER/Line Shapefiles) dataset using geographic identifiers (GEOIDs) (US Census Bureau, 2022a, 2022b; 2022c; 2022d; 2022e, n.d.). We calculated adult population density using the ACS of the adult population with land area data from the TIGER files.

Urbanicity was assigned using the National Center for Education Statistics (NCES) locale classification, which defines four categories: city, suburban, town, and rural (NCES, n.d.). Following prior research, we combined the town and suburban categories into a single suburban category and referred to city areas as urban. This standardized classification provides a nationally consistent framework based on population size and proximity to metropolitan centers (Slotman et al., 2022, 2022, 2022).

In addition, we used the 2019 edition of the Social Deprivation Index (SDI), which measures socioeconomic deprivation at the census-tract level through seven socioeconomic indicators (Robert Graham Center, 2018). We selected the SDI over other deprivation indices because it accounts for households without vehicles —a direct measure of vulnerability related to transportation access— and does not include community-level race and ethnicity, allowing us to examine these variables independently. The SDI provides a percentile rank for each census tract, ranging from 1 (least deprived) to 100 (most deprived) (Robert Graham Center, 2018).

To represent racial and ethnic segregation, we used the dissimilarity index, which quantifies the evenness of population distribution between racial or ethnic groups across geographic units. We calculated two indices, Black–White and Hispanic–White, using 2022 ACS data, consistent with prior national segregation studies (Chambers et al., 2018). The highest value of this index (1) indicates complete segregation, while the lowest (0) indicates complete integration (Chambers et al., 2018). These two comparisons capture the majority of racial and ethnic residential segregation in the U.S., as approximately 88.7% of the population identifies as non-Hispanic White, non-Hispanic Black, or Hispanic (US Census Bureau, 2022f). Populations outside these categories were not included in segregation calculations due to their small size and limited reliability at the block-group level.

### 2.4 Statistical analyses

First, we examined variations in travel times to the nearest dental clinic at the block group level across urban, suburban, and rural areas and compared these variations among the nine U.S. Census Divisions. We also compared the percentage of adult population living within a 10-min drive and a 30-min public transit ride to dental care across states within each census division.

There is no universally accepted travel-time threshold for defining access to dental clinics. We therefore categorized access into three levels: extensive, moderate, and low, using cutoffs at 10 min and 30 min, informed by several considerations. First, the data distribution suggested 10 min as an empirical upper bound for extensive access. Second, 10 min is a widely recognized benchmark for emergency medical response by the National Association of State Emergency Medical Service Officials (Choi et al., 2022; Clark et al., 2025). Third, international evidence indicates that more than 60% of the global population lives within 10 min of a medical clinic (Weiss et al., 2020). Fourth, prior health service research commonly considers less than 30 min of travel to medical care as indicative of good access, whereas longer travel times are associated with transportation burden (Probst et al., 2007; Rahman et al., 2024). Finally, the U.S. Department of Veterans Affairs uses a 30-min drive time standard for defining appropriate access to dental care (Veterans Affairs, 2019). Using data from the Google Maps Distance Matrix API, we calculated the percentage of the population in each state without public-transit access (Google, 2024a) and compared the mean drive times for block groups with and without access to public transit, examining the distribution of travel times across urbanicity categories.

Next, we used the Getis-Ord Gi∗ statistics to identify spatial clusters (hotspots) of social deprivation and travel time to the nearest dental clinic (Getis & Ord, 1992). Clusters were evaluated at the 95% confidence level using the K-nearest-neighbor spatial relationships with 30 nearest neighbors per block group (ESRI, n.d.). We generated bivariate maps to visualize the relationship between SDI hotspots and travel times. Block groups vary substantially in size and shape; therefore, a k-nearest-neighbor approach provides a consistent number of neighbors for each observation. Low *k* values generated fragmented clusters, while high values over-smoothed local variations. Recent health and transportation studies commonly use k values between 4 and 60 (Czepkiewicz et al., 2024; Jackson et al., 2009; Wong et al., 2024). Because each census tract contains, on average, about 3 block groups and spatial density varies across regions, we selected k = 30 as a mid to upper range value, capturing neighbors across approximately eight to ten census tracts. Longer travel-times hotspots, defined by a GI∗ z-score ≥1.96, indicate regions where residents experience significantly prolonged travel times, often exceeding 31.8 min in public transit and 15.3 min for driving.

Lastly, we conducted two separate spatial lag logistic regression models for drive time and public transit time, stratified by neighborhood type (urban, suburban, and rural), to examine the characteristics of block groups associated with longer travel time hotspots. We created binary outcome variables for longer travel-time hotspots (Gi∗ z-score ≥1.96 = 1; non-hotspot = 0). Our outcome variable is a hotspot, which is characterized by longer travel time clusters. Therefore, to account for spatial dependency, we incorporated a spatially lagged independent variable and outcome variables using a first-order queen contiguity spatial weight matrix. This spatial lag framework allows for the simultaneous estimation of both local effects and spillover effects of neighboring block groups. Both models were estimated at the block group level, with six independent variables: neighborhood types, adult population density, the percentage of uninsured adults, Black and Hispanic segregation, and SDI. The percentage of uninsured adults and population density were standardized (z-score normalization). High-levels of segregation for Black and Hispanic populations were defined as the 4th quartile of the dissimilarity index, with the 1st to 3rd quartiles as the reference group. For SDI, the first tertile (least deprived) served as the reference group.

We evaluated model performance using AIC, BIC, loglikelihood, deviance, and McFadden's pseudo-R^2^. All models for drive time demonstrate a strong fit, with McFadden's pseudo-R^2^ values (overall = 0.88, stratified suburban = 0.93, and stratified rural 0.77) indicating substantial explanatory power (Table S3). High R^2^ and low deviance indicate that the spatial-lag logistic models effectively compute geographic and sociodemographic patterns associated with drive time hotspots.

Similarly, overall public transit models and stratified public transit models indicate an excellent fit for the spatial lag model. The overall model for public transit has a high R^2^ (0.84) value along with a reasonable AIC value of 27,305.6 for a large data set (Table S4). Stratified urban, suburban, and rural models for public transportation have R^2^ 0.86, 0.86, and 0.69, respectively.

## 3 Results

### 3.1 Travel times to the nearest dental clinic by driving and public transport

**Fig. 1:**
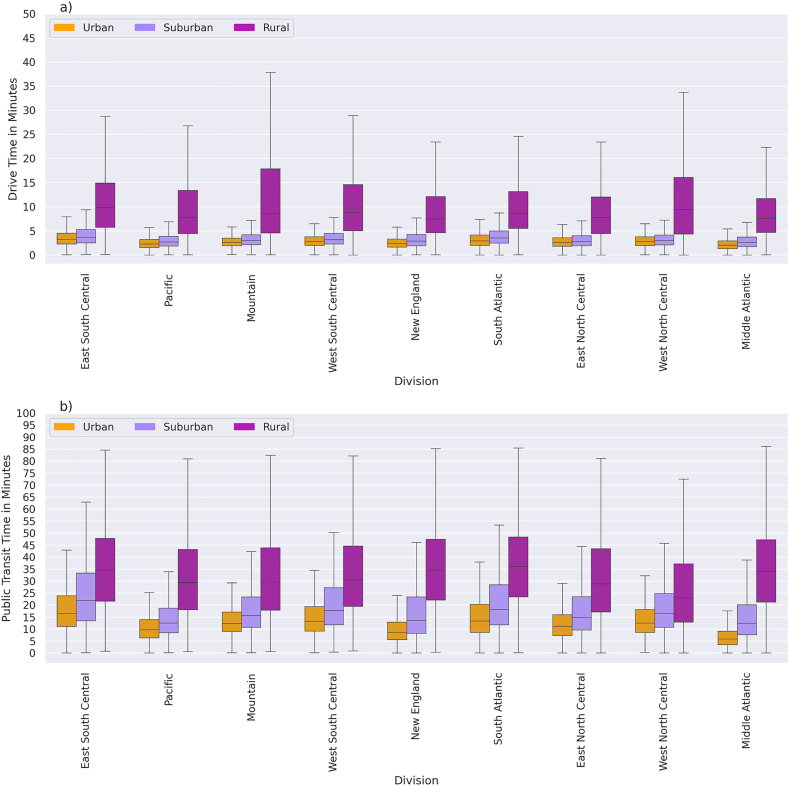
Travel time distribution to the nearest dental clinics in urban, suburban, and rural areas by the census divisions. **Note.** Travel time calculation to adult dental clinics. Authors' analysis of data from 1) Dentists database from IQVIA; the categories of Urban suburban, and rural are based on urban, suburban, and rural block groups. a) drive time and b) public transit time. The number of observations and descriptive statistics are in supplementary appendix tables (S1 and S2).

The national mean driving time to the nearest dental clinic was 5.8 min (SD = 5.9) across all block groups. The mean (SD) driving times in urban, suburban, and rural areas were 2.8 (1.5), 3.4 (2.2), and 10.5 (8.7) minutes, respectively. Our analysis shows that almost no adults in urban and suburban areas need to drive more than 10 min to reach the nearest dental clinic (Fig. 1a). Areas with short drive times (≤10 min) were concentrated in the Mid-Atlantic and Northeastern Central states, whereas rural areas in the Mountain and West North Central divisions experience extended driving times exceeding 30 min (Fig. 1a, Fig. S2). In sensitivity analyses conducted for California and Montana, mean driving times calculated at several alternative off-peak hours (8 a.m., 9 a.m., 1 p.m., 3 p.m., and 4 p.m.) varied by less than 0.1 min in Montana and less than 0.15 min in California, indicating that the choice of reference time within typical off-peak periods had a negligible influence on estimated travel times. Moreover, Cohen's d and Bland-Altman analysis demonstrated excellent agreement between the 10:00 a.m. and 4:00 p.m. travel time estimates in both California (Cohen's d = 0.43, r = 0.99, and mean difference = 0.09 min) and Montana (Cohen's d = −0.07, r = 0.99, and mean difference = 0.02 min). The results confirm that 10:00 a.m. travel times provide a robust and representative measure of baseline dental travel accessibility.

Although population density differs between suburban areas and small towns (2009 vs. 1017 people), travel times to the nearest dental clinic are similar. Mean drive times are 3.3 and 3.6 min, respectively, and average public transit times are 18.4 and 18.8 min. Given these comparable travel time patterns, we combined suburban areas and small towns into a single suburban category for subsequent analyses and discussion.

The national mean public transit time to the nearest dental clinic is 18.1(SD = 18.3) minutes in areas where public transit was available. The mean (SD) times in urban, suburban, and rural areas were 13.0 (9.5), 18.5 (13.7), and 34.3 (37.8) minutes, respectively. Unlike driving times, public transit times were substantially longer in suburban than in urban areas (Fig. 1b, Fig. S3). In most census divisions, the median transit time in rural areas exceeded 30 min, while it was under 15 min in urban areas (Fig. 1b). The difference in mean driving time between areas with and without access to public transit was greatest in rural areas (10.3 min) compared with 4.2 min in urban and 3.7 min in suburban areas (Fig. S4).

### 3.2 Distribution of population by drive time and public transit across census divisions

**Fig. 2:**
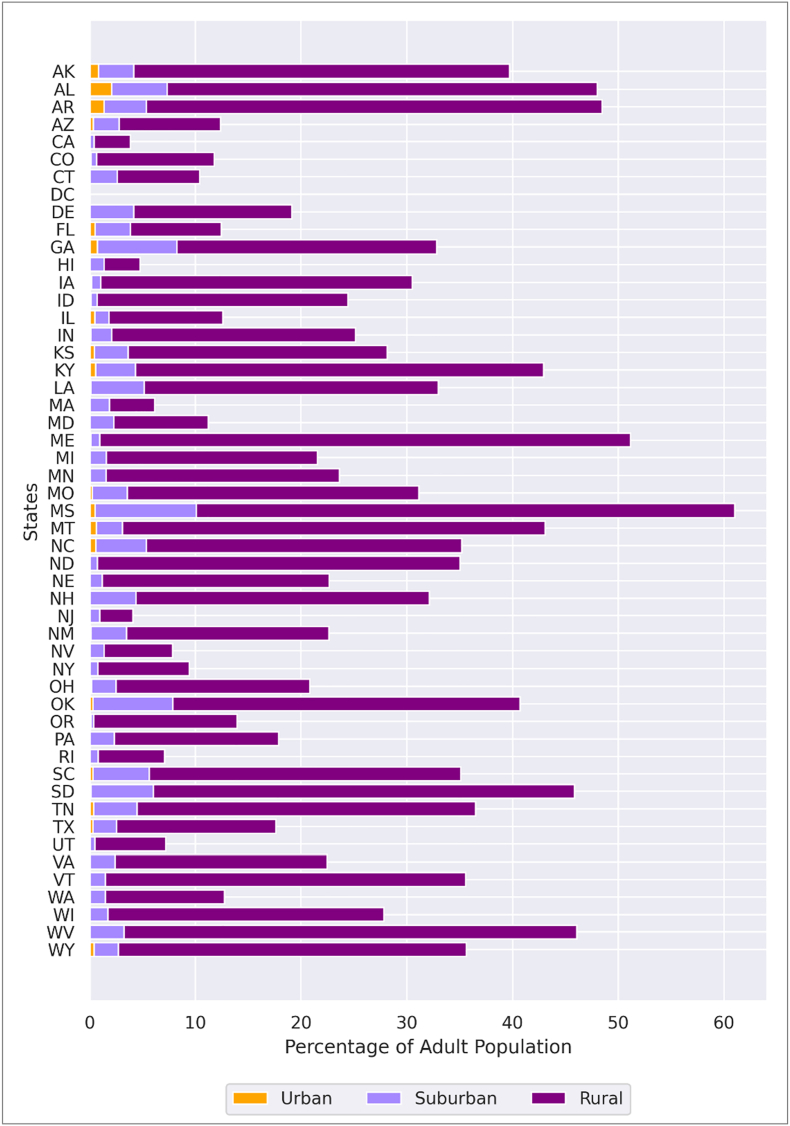
Percentage of adult population without access to public transit to the nearest dental clinic. **Note.** Public transit time calculation to adult dental clinics. Authors' analysis of data from 1) Dentists' database from IQVIA; 2) American Community Survey 2022 estimates of the population from US Census Bureau. Population without access to public transit refers to the population in block groups where public transit is unavailable, according to Google Maps API query.

Approximately 49.3 million (19.1%) of U.S. adults live in block groups where public transit is unavailable to reach the nearest dental clinic. These block groups correspond to origins for which the Google Maps Distance Matrix API returned “Zero_Results,” indicating no valid public transit route between the block-group population mean center and the nearest clinic. There are 0.6 million urban adults (1.3%), 6.2 million suburban adults (12.5%), and 42.5 million rural adults (86.2%) without access to public transit. More than half of adults in Missouri (61.0%) and Maine (51.1%) lack public transit access, whereas all adults in Washington, D.C., have access (Fig. 2). About 172.5 million (66.9%) adults can reach the nearest dental clinic within 30 min by public transit, while 36.1 million (14.0%) face extended transit time (>30 min). The Pacific Division has the highest percentage of adults (85.0%) with access within 30 min using public transit, whereas, East South Central has the lowest percentage (37.9%) (Fig. S).

About 231.8 million (89.9%) adults can reach the nearest dental clinic within a 10-min drive. The Pacific Division has the highest percentage of adults with access within a 10-min (94.9%), while East South-Central Division has the lowest (77.1%) (Fig. S6).

### 3.3 Travel times and social deprivation hotspots

**Fig. 3:**
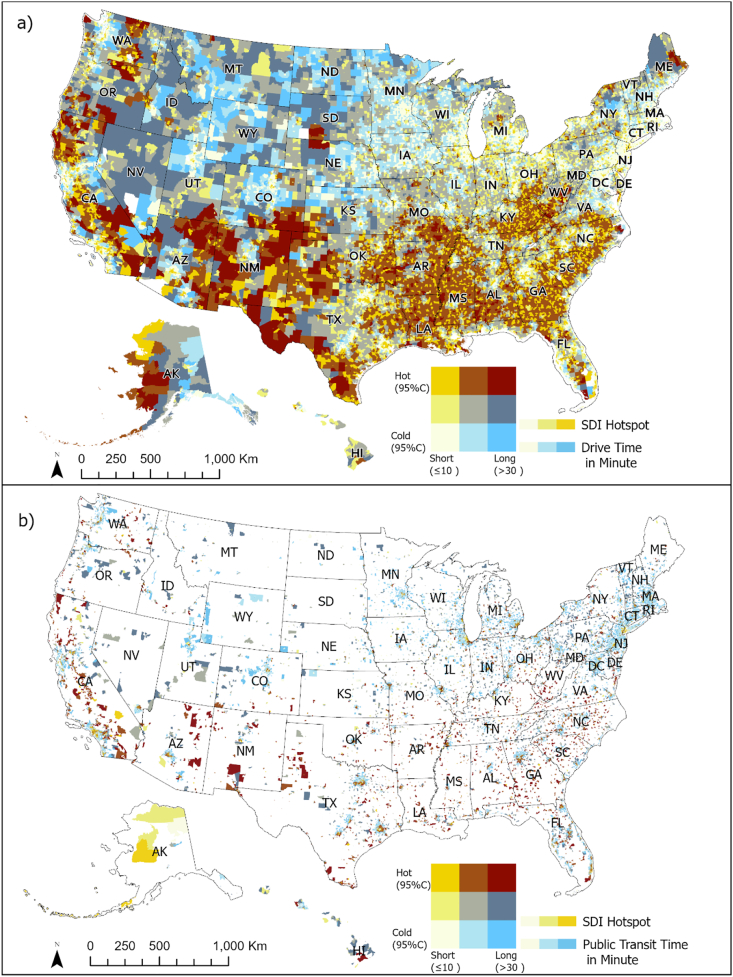
Bivariate maps of the hotspots of the Social Deprivation Index (SDI) and travel time to the nearest dental clinic. **Note.** Travel time calculation to adult dental clinics. Authors' analysis of data from 1) Dentists database from IQVIA; 2) American Community Survey 2022 estimates of population and the TIGER/line shapefiles from US Census Bureau. a) Bivariate representation of SDI hotspot and drive time to the nearest adult dental clinic in minutes. b) Bivariate representation of SDI hotspot and public transit travel time to the nearest adult dental clinic in minutes. Categories (a 3 by 3 legend scheme) are based on the Gi∗ score of SDI, divided at −1.96 and + 1.96 to identify hotspots and cold spots at 95% confidence intervals, with travel times split at 10 min and 30 min, respectively. SDI hotspots and cold spots at 95% confidence level; Short and long travel time are categorized as less than or equal to 10 min and greater than or equal to 30 min, respectively. The whitespace indicates areas lacking data on public transit, signifying block groups where public transit is unavailable according to the Google Maps API query.

The dark red areas in Fig. 3 indicate regions with high social deprivation where residents experience travel times over 30 min to reach the nearest dental clinic. Fig. 3a shows that block groups identified as SDI hotspots with extended drive time (>30 min) are primarily located in Texas, New Mexico, Arizona, and California.

Public transit is unavailable to nearly 49.3 million (19.1%) of U.S. adults. Among those with public transit access, 74.5 million adults (35.7%) reside in SDI hotspots. Similar to driving times, SDI hotspots associated with public transit travel burdens over 30 min are concentrated in Southern states (Fig. 3b).

### 3.4 Characteristics of hotspots for driving and public transit times

The mean travel time in public transit hotspots was 31.8 min, compared to 15.3 min in non-hotspot areas. Similarly, the mean driving times are 12.4 min and 3.8 min in hotspot and non-hotspots areas.

In overall spatial lag regression model for drive-time hotspots, rural and suburban block groups were significantly more likely to be classified as hotspots compared with urban block groups (Table S5). Areas with extreme Black and Hispanic segregation, and high social deprivation, were also more likely to be in drive-time hotspots. For example, Black-segregated and Hispanic-segregated block groups were 1.2 times (95% CI: 1.1–1.3) and 1.1 times (95% CI: 1.0–1.2) more likely, respectively, to be drive-time hotspots compared with less segregated areas. In contrast, areas with a high density of the adult population were 0.1 times (95% CI: 0.0–0.3) less likely in drive time hotspots.

The large odds ratio for the spatial lag of the dependent variable indicates strong spatial clustering of travel time hotspots, suggesting that these clusters are geographically contiguous rather than isolated block groups (Table S6). In other words, block groups are more likely to be classified as hotspots when neighboring block groups are also hotspots, reflecting spatial dependence and spillover effects in accessibility.

Stratified regression analyses for rural areas showed that Black-segregated block groups were 1.1 times (95% CI: 1.1–1.3) and Hispanic-segregated block groups were 1.1 times (95% CI: 1.0–1.2) more likely to be drive-time hotspots as non-segregated block groups (see Table 1). The most deprived block groups were substantially more likely to be in hotspots, with odds 1.7 times higher in rural areas (95% CI: 1.2–1.5) and 2.1 times higher in suburban areas (95% CI: 1.2–3.7) compared with the least deprived areas (see Table 1).

**Table 1:** Characteristics of hotspots for driving and public transit times across urban, suburban, and rural block groups.

	Drive Time Hotspots			Public Transit Time Hotspots		
	*Urban*^*a*^	*Suburban*^*b*^	*Rural*^*c*^	*Urban*^*a*^	*Suburban*^*b*^	*Rural*^*c*^
**Total number of block groups**	37	5601	32,871	2936	18,603	10,794
**Mean travel time in minutes**	2.8	3.4	10.5	13.0	18.5	34.3
	Odd Ratio [95% CI]					
**Adult Population Density**		0.3∗ [0.1, 1.1]	0.00∗∗∗∗ [0.00, 0.00]	0.2∗∗∗∗ [0.1, 0.4]	0.4∗∗∗∗ [0.2, 0.6]	0.00∗∗∗∗ [0.00, 0.00]
**Uninsured adult population (%)**		0.9 [0.8, 1.1]	1.0 [0.9, 1.0]	1.0 [0.9, 1.1]	1.0 [0.9, 1.1]	1.0 [0.9, 1.1]
**Black segregation**						
Dissimilarity index (1st to 3rd quartile)	Reference	Reference	RReference	Reference	Reference	Reference
Dissimilarity index (4th quartile)		1.2 [0.9, 1.5]	1.1∗∗∗ [1.1, 1.3]	1.0 [0.7, 1.5]	0.9 [0.8, 1.1]	0.9 [0.8, 1.0]
**Hispanic segregation**						
Dissimilarity index (1st to 3rd quartile)	Reference	Reference	Reference	Reference	Reference	Reference
Dissimilarity index (4th quartile)		1.1 [0.8, 1.5]	1.1∗∗ [1.0, 1.2]	1.0 [0.7, 1.4]	1.1 [0.9, 1.3]	0.9∗ [0.8, 0.1.0]
**Social Deprivation Index (SDI)**						
SDI-1 (least deprived)	RReference	Reference	Reference	Reference	Reference	Reference
SDI-2		1.7∗∗∗ [1.2, 2.5]	1.3∗∗∗∗ [1.2, 1.5]	0.9 [0.7, 1.2]	1.1 [0.9, 1.2]	1.9∗∗∗∗ [1.6, 2.2]
SDI-3 (most deprived)		2.1∗∗∗ [1.2, 3.7]	1.7∗∗∗∗ [1.4, 2.0]	0.8 [0.5, 1.2]	1.2 [0.9, 1.5]	3.0∗∗∗∗ [2.4, 3.8]

**Note.** Travel time calculation to adult dental clinics. Authors' analysis of data from 1) Dentists database from IQVIA; 2) American Community Survey 2022 estimates of population and the TIGER/line shapefiles from US Census Bureau. ^a^ Only urban block groups; ^b^ Only suburban block groups; ^c^ Only rural block groups. The regression model for drive time hotspots in urban areas could not be estimated due to the small number of available hotspot block groups.∗p < 0.10, ∗∗p < 0.05, ∗∗∗p < 0.01 ∗∗∗∗p < 0.001. 3) Model fit information is provided in the supplementary appendix (Table S3 and S4).

For transit-time hotspots, rural and suburban block groups were also significantly more likely than urban areas to be hotspots (Table S5). In contrast, block groups with the highest levels of social deprivation had substantially higher odds (OR = 3.7; 95% CI: 3.2–4.3) of being hotspots than the least deprived areas. In addition, Hispanic segregated areas were 1.2 times (95% CI: 1.0–1.3) more likely in public transit hotspots.

Hispanic-segregated rural block groups were 0.9 times (95% CI: 0.8–1.0) less likely to be transit hotspots (see Table 1). The most socially deprived block groups in rural areas were 3.0 times (95% CI: 2.4–3.8) more likely to be hotspots compared with their least deprived counterparts (see Table 1).

## 4 Discussion

There are significant disparities in travel times to access dental care, with variations across different regions and demographic groups (Rahman et al., 2024). About 49.3 million (19.1%) U.S. adults lack public transit access, and an additional 36.1 million (14.0%) face extended travel burdens (>30 min) to reach the nearest dental clinic. Moreover, areas with lower population density and a higher percentage of uninsured adults are more likely to be hotspots for both driving and public transit, regardless of whether they are urban, suburban, or rural. These findings expand upon earlier research that has documented disparities in the geographic distribution of dental providers (Rahman et al., 2024) by quantifying, for the first time at a national scale, how both driving and public-transit travel times vary across social and spatial contexts.

Although public transit coverage is limited across many regions, especially in suburban and rural areas, this limitation is itself a central finding. The absence of available routes represents a form of transportation inequity that restricts the mobility of populations who rely on public transit for accessing healthcare. By examining both where transit access exists and where it is absent, our analysis captures travel-time burdens and the systemic exclusion of entire communities from transit-based access to dental care. This framing emphasizes public transit accessibility as both an indicator of existing and potential access to care. This dual focus on presence and absence of transit networks provides a more comprehensive view of transportation inequities than previous studies, which have largely focused on either provider density or driving accessibility alone.

Historically, racial and ethnic minority individuals and those with low incomes have faced various forms of socioeconomic deprivation, including limited access to healthcare. The 1968 Fair Housing Act, which abolished redlining, and the great migration of rural Black populations to major cities, significantly altered the demographic and structural landscape of urban and suburban neighborhoods (Dilworth & Gardner, 2019; Tolnay, 2003). As a result, these areas experienced significant demographic shifts, with wealthier, predominantly White residents moving to suburban areas and Black populations moving into cities. This shift in demographic composition, particularly in metropolitan regions, has contributed to disparities in access to resources and opportunities. For example, transit-oriented development, which was intended to enhance accessibility, has often been implemented in ways that reinforce existing social and economic inequalities (Li et al., 2023).

In suburban and rural areas, block groups with extreme social deprivation and high levels of Black and Hispanic segregation were more likely to be in drive time hotspots, possibly as a result of decades of transportation planning that prioritized the needs of White populations over minoritized populations (Bloom, 2023). In large cities, transit-oriented developments, such as high-density, mixed-use complexes near metro or rail-based public transport, tend to attract residents who cannot afford personal vehicles, despite high rents (Deka, 2017; Keeler & Stephens, 2023; Oh & Wang, 2018; Tolnay, 2003). As a result, block groups with extreme social deprivation are less likely to be found in public transit hotspots in urban and suburban contexts but are more prevalent in rural areas. This is particularly evident in rural Black-segregated block groups, which are less likely to be in public transit hotspots, as 14.7% (5.8 million) of Black individuals live in rural areas compared to 45.7% (18.2 million) in urban and 39.6% (15.8 million) suburban areas, and the general lack of public transportation infrastructure in these areas.

Despite being more dependent on public transportation than White individuals, racial and ethnic minorities and low-income populations face persistent inequities in accessing dental care due to the systematic underfunding of public transit systems since the 1970s. Transit equity remains a pressing issue that affects not only to healthcare but also opportunities for to employment, education, and essential services (Akinlotan et al., 2023; Anderson, 2016; Bloom, 2023; Javid & Sadeghvaziri, 2022; Pucher & Renne, 2003). Our results contribute to this growing field of transit equity research by providing empirical evidence that transportation disadvantage remains a measurable and quantifiable determinant of oral healthcare access across the United States.

Our analysis indicates that nearly 14 million adults living in areas of high social deprivation lack access to public transit. Many households in these socially deprived block groups do not own a vehicle (Robert Graham Center, 2018), further compounding their transportation challenges when public transit is also unavailable (Scaturro, 2024). Moreover, 86.2% of adults who lack access to public transit (49.3 million) reside in rural areas, where mean drive times are longer than in urban and suburban areas, posing an additional financial and time burden. Adults without public transit access often face longer drives to the nearest dental clinic, regardless of urbanicity, compared to those with public transit access. These transportation barriers can lead to missed or delayed dental appointments, resulting in poor oral health and higher healthcare costs, particularly for older adults and individuals with disabilities who may be unable to drive (Heaps et al., 2021; Scaturro, 2024; Smith et al., 2022). Expanding nonemergency medical transportation (NEMT) benefits within public and private dental plans may help reduce transportation barriers and improve the accessibility for low-income, racial and ethnic minorities, socioeconomically deprived, and rural adults (Shen et al., 2024).

Residents of large metropolitan areas generally have access to more public transportation options, including metro, train, and bus services, while smaller cities often rely solely on bus services.^41^ Comparing public transit travel times to the nearest dental clinic across major metropolitan areas with multimodal transit systems could provide valuable insights into how transit infrastructure affects access to dental care across different urban settings.

## 5 Limitation

First, our measure of public transit availability is based on Google Maps routing data for a single weekday and reflects access only to the nearest (shortest travel time) clinic. Therefore, only 1.4% of block groups where public transit was available classified as lacking transit access to the nearest clinic (shortest distance) may still have routes to more distant ones. Second, we examined travel times to the nearest dental clinic, but individuals may bypass the nearest clinic for reasons such as insurance acceptance, urgency of care, or appointment availability. Moreover, this study focused on spatial accessibility measured by travel time and did not account for clinic service capacity or population demand. Future research should integrate both supply- and demand-side measures, such as the number of dentists, population served, appointment availability, and insurance participation, to provide a more comprehensive assessment of access. Third, our reliance on the Google Maps Distance Matrix API to identify public transit unavailability may not fully capture the complexity of existing transit options. Further, “Zero_Results” returns from public transit queries on the Google Maps Distance Matrix API may reflect multiple conditions, including unreasonable walking distance to the nearest transit stop at the queried time or the absence of public transit networks. Therefore, our estimates may underestimate the actual travel burden in affected areas. Fourth, drive time and public transit time were calculated using different platforms, ESRI ArcGIS for driving and Google Maps for transit, which use distinct routing algorithms and data sources. This may introduce some inconsistency between the two measures. However, because our objective was not to directly compare driving and transit times, we analyzed and interpreted them independently, avoiding cross-modal comparisons that could be influenced by these differences. Fifth, there is heterogeneity within the suburban category, where we combined small towns and suburban classes into one group. Sixth, while we calculated travel time from the population mean center to represent where most residents are located, rural block groups are larger and more sparsely populated than urban ones, which may reduce precision. Depending on block-group size and spatial population distribution, individual travel times may vary by several minutes within the same block group. Travel times were also calculated for an off-peak weekday (Tuesday at 10:00 a.m.), which may not reflect rush hours or weekend conditions; however, sensitivity analyses in two contrasting states showed minimal variation (<0.15 min) across several off-peak weekday times. Seventh, our analysis focused on access to routine and preventive dental services rather than specialized dental care. Lastly, our analysis assumed that the resident adult population within each block group represents the potential population in need of routine dental care. Although this approach aligns with prior studies of spatial accessibility, it does not account for variation in utilization patterns or unmet need across demographic groups. Future research could extend this framework by integrating measures of demand or clinical utilization.

## 6 Conclusions

Our analysis highlights significant disparities in travel times to the nearest dental clinic across urban, suburban, and rural areas. We found that a substantial proportion of the U.S. population lacks access to public transit, creating additional barriers for vulnerable groups in accessing necessary dental care. Addressing these transportation barriers will require better alignment between transportation and healthcare policies to improve public transit systems, incentivize dentists to practice in underserved rural areas, expand NEMT benefits within public and private insurance programs, and increase the availability of mobile dental clinics (Dawkins et al., 2013; Lehnert & Thakur, 2024). Areas identified as travel time hotspots with high social deprivation should be prioritized for the allocation of mobile dental units, teledentistry infrastructure, and incentives to establish new clinics, including initial financial support and tax exemptions (Nichols, 2019; Ward, M. M. et al., 2022). Because transportation barriers differ by community type, tailored policy interventions are likely to be most effective. In urban areas, policies should emphasize transit route optimization, micro-transit systems, and on-demand shuttle services. In rural areas, efforts should focus on increasing the number of dental clinics through targeted loan-repayment programs for early-career dentists, subsidies for clinic establishment, state-supported satellite clinics, and deployment of mobile care units. These strategies can help mitigate transportation barriers that disproportionately affect vulnerable populations, promote transit equity, and enhance equitable access to dental care nationwide.

## CRediT authorship contribution statement

**Md Shahinoor Rahman:** Writing – review & editing, Writing – original draft, Visualization, Methodology, Formal analysis, Data curation. **Ichiro Kawachi:** Writing – review & editing, Validation, Methodology, Investigation, Funding acquisition, Conceptualization. **Benjamin D. Sommers:** Writing – review & editing, Validation, Methodology, Investigation. **Renuka Tipirneni:** Writing – review & editing, Validation, Funding acquisition, Conceptualization. **Jeffrey C. Blossom:** Writing – review & editing, Methodology, Investigation, Formal analysis. **Hawazin W. Elani:** Writing – review & editing, Writing – original draft, Validation, Supervision, Resources, Project administration, Methodology, Investigation, Funding acquisition, Data curation, Conceptualization.

## Ethical statement

This cross-sectional study was determined to be not human participants research and therefore did not require informed consent by the institutional review board of the Harvard Faculty of Medicine.

## Funding/Support

This work was supported by award R01MD017093 from the National Institute on Minority Health and Health Disparities. The National Institute on Minority Health and Health Disparities had no role in the design and conduct of the study; collection, management, analysis, and interpretation of the data; preparation, review, or approval of the manuscript; and decision to submit the manuscript for publication. This article does not reflect the views of the National Institutes of Health, the US Department of Health and Human Services, or the US government.

## Declaration of interests

The authors declare that they have no known competing financial interests or personal relationships that could have appeared to influence the work reported in this paper.

The authors declare the following financial interests/personal relationships which may be considered as potential competing interests:

Hawazin W. Elani reports financial support was provided by National Institute on Minority Health and Health Disparities. Md Shahinoor Rahman reports financial support was provided by National Institute on Minority Health and Health Disparities. Ichiro Kawachi reports financial support was provided by National Institute on Minority Health and Health Disparities. Renuka Tipirneni reports financial support was provided by National Institute on Minority Health and Health Disparities. If there are other authors, they declare that they have no known competing financial interests or personal relationships that could have appeared to influence the work reported in this paper.

## Data Availability

The authors do not have permission to share data.
