# Supplementary material for: Driving and public transit barriers to dental care in the United States
Source: SSM Popul Health. 2026 Mar 10;33:101907. doi: 10.1016/j.ssmph.2026.101907 (PMC12996270; doi:10.1016/j.ssmph.2026.101907)
Supplement: Multimedia component 1 [file mmc1.docx]

**Driving and public transit barriers to dental care in the United States**

**Appendix.** Methods.

**Figure S1.** The cumulative percentage of sampled block groups and the rank of the clinic with the shortest travel time via public transit.

**Figure S2**. Drive time to the nearest dental clinics.

**Figure S3**. Public transit time to the nearest dental clinics.

**Figure S4.** The difference in mean drive time between block groups with and without public transit.

**Figure S5.** Cumulative population distribution by public transit time across census divisions

**Figure S6.** Cumulative population distribution by drive time across census divisions

**Table S1.** Descriptive statistics of drive time by census division in the US

**Table S2.** Descriptive statistics of public transit time by census division in the US

**Table S3.** Model fit indices for the spatial lag regression models for drive time

**Table S4.** Model fit indices for the spatial lag regression models for public transit

**Table S5.** Characteristics of hotspots for driving and public transit times.

**Table S6.** Lag odd for the spatial lag regression models for driving and public transit times

**Appendix- Methods**

**Mapping dental clinics**

To calculate the travel time from the population center of block groups to the nearest dental clinics, we need the precise location of the clinics. In our study, a clinic location refers to all dental practices sharing the same street address. IQIVIA database, assigns each street address a unique Street Front ID. IQIVIA data is based on numerous government, non, non-government, and industry sources and undergoes rigorous cleaning, updating, and validation through medical and prescription claims, telephone calls to clinics, web searches, and verification with state boards (IQIVIA, 2023).

For our analysis, we aggregated individual practitioners into 104,695 clinic locations using the Street Front ID. We then geocoded each clinic location (i.e., obtained latitude and longitude) using ArcGIS World Geocoding Services.(ESRI, 2012) The final dataset includes the Street Front ID for each location along with its coordinates, total number of dentists, specialty breakdown, gender, and Medicare acceptance status. These aggregated data enable travel time calculations between block group population centers (origins) and clinic locations (destinations).

**Calculating Dissimilarly Index**

We used the dissimilarity index, an evenness measure of segregation, to assess the distribution of Non-Hispanic Black and Hispanic populations relative to White populations at the block group level. The dissimilarity index represents the proportion of a particular race/ethnic group that would have to move to achieve an even distribution of the White population (Chambers et al., 2018).

We obtained race data at the block group level from the ACS estimation of 2022. (US Census Bureau, 2022a) We calculated the dissimilarity index using the following equation.


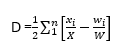


D is the dissimilarity index, where the scores range from zero (complete integration) to one (complete segregation). x_i_ is the population of the targeted race (e.g. Black and Hispanic) and w_i_ is the White population in a block group. X and W represent the total selected race population and total White population at the census tract of the corresponding block group.

**Mapping population center**

We calculated travel times between dental clinics and block group population centers, defined as population-weighted mean centers. Because census blocks nest within block groups, we obtained the necessary demographic data at the census block level from the U.S. Decennial Census 2020 (US Census Bureau, 2022b).

First, we calculated the geometric center of each block based using its polygon boundaries. Next, we determined the population-weighted mean center for each block group by averaging the geometric centers (latitudes and longitudes) of all blocks within that group, weighted by each block’s population. In cases where a block group had no population, we used its geometric center rather than a population-weighted mean to avoid a zero value in the calculation. Finally, we adjusted (or “snapped”) each population mean center to the nearest road network segment to avoid placing origins in impractical locations, such as the middle of a lake.

**Drive time measurement**

To determine travel times and distances between block group population centers and dental clinics, we used the ArcGIS Pro origin-destination cost matrix tool (ESRI, n.d.). We calculated drive times and distances from each population center to all clinics within a three-hour travel radius. We then filtered these results to identify the shortest drive time per block group, to determine the nearest clinic.

The three-hour threshold was chosen to balance computation time with data completeness. Preliminary analysis indicated that every block group population center has at least one clinic within a three-hour drive, making this a practical cut-off for effectively managing large datasets without sacrificing accuracy.

**Public transit time measurement**

Because requests to the Google Distance Matrix API are costly, we sought to minimize the number of public transit travel time calculations (Google, 2024). We limited each block group’s query to the 17 geographically closest clinics (i.e., by straight-line distance). To validate this cut-off, we randomly sampled 2,392 block groups (representing 1% of all block groups), with half drawn from rural and half from urban counties. For each sampled block group, we calculated public transit travel times to nearby clinics and plotted the frequency of block groups against each clinic’s distance rank (see Figure S1).

The analysis revealed that, for all sampled block groups, the clinic with the shortest public transit travel time was within the 15th closest clinic by straight-line distance. To ensure a margin of safety (10%), we selected 17 as the optimum number of clinics to query per block group. Finally, for each block group, the clinic with the shortest travel time (from among the 17 queried) was deemed the nearest clinic (Google, 2024).

**Figure S1.** The cumulative percentage of sampled block groups and the rank of the clinic with the shortest travel time via public transit


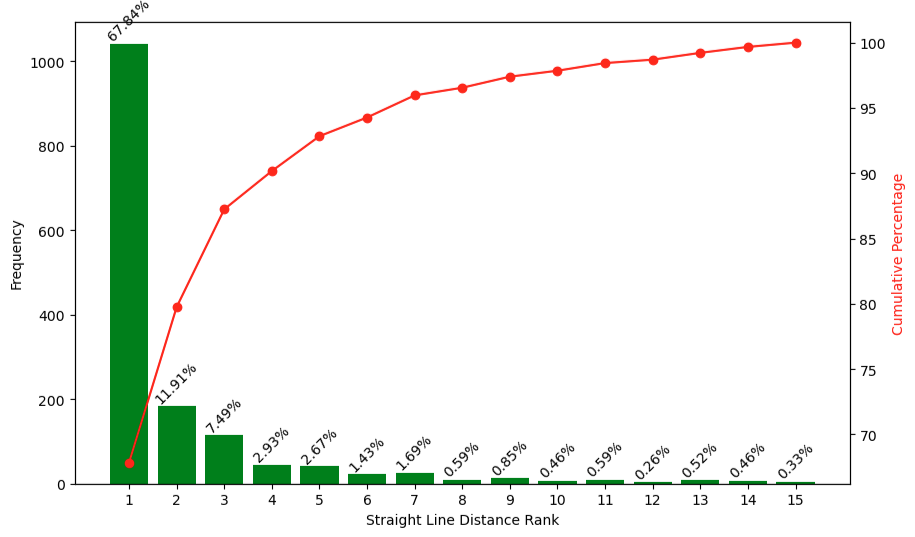


**Note.** Travel time calculation with public transit to adult dental clinics. Authors’ analysis of data from 1) Dentist database from IQVIA; 2) Travel time calculated using the Google Distance Matrix API.

**Figure S2.** Drive time to the nearest dental clinics


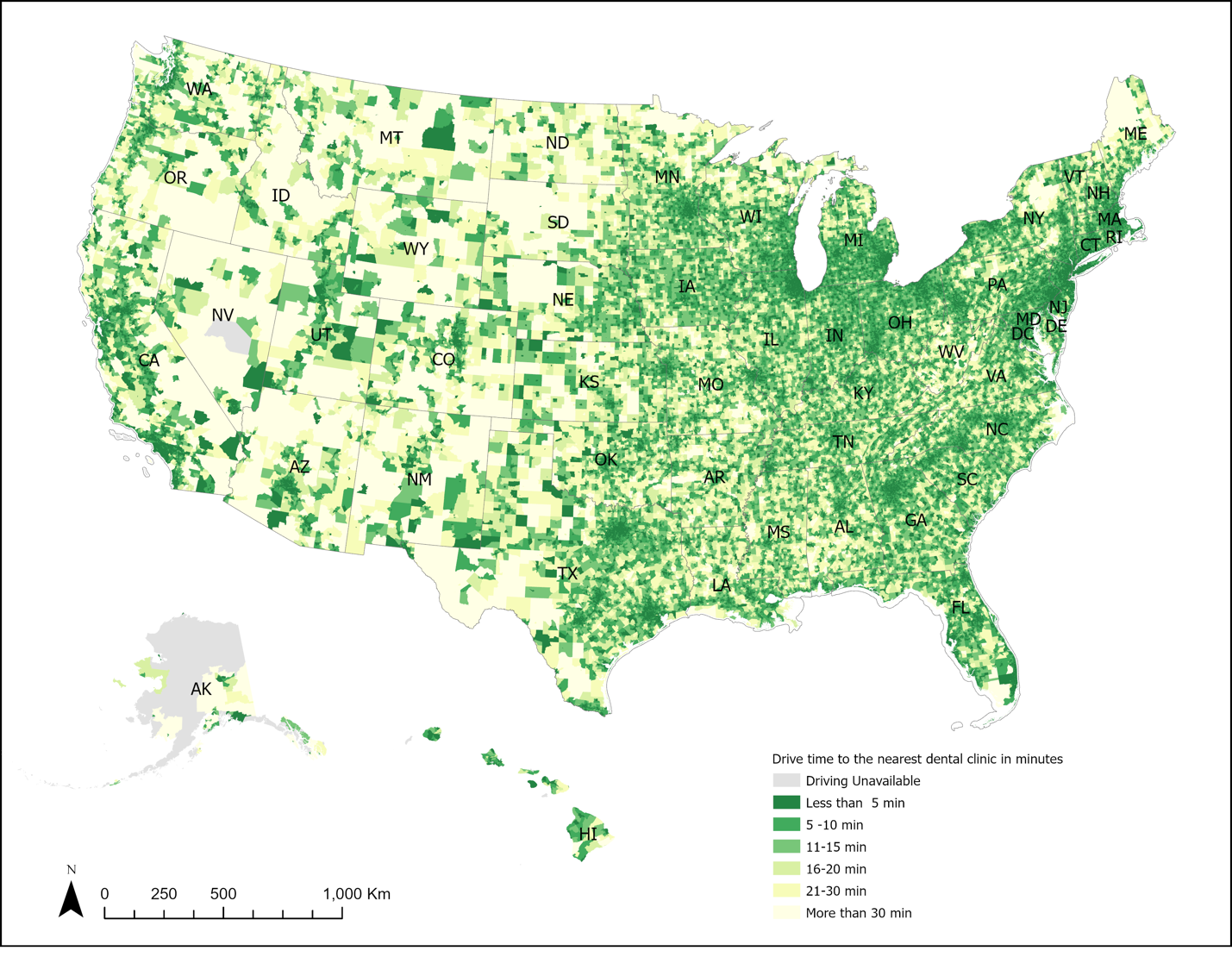
**Note.** Drive time calculation to adult dental clinics. Authors’ analysis of data from 1) Dentist database from IQVIA; 2) American Community Survey 2022 estimates of population and the TIGER /line shapefiles from US Census Bureau. Categories are based on drive time in minutes from the block group population center to the nearest dental clinics. Drive time unavailability means block group centers do not have road networks within 30 miles.

**Figure S3.** Public transit time to the nearest dental clinics


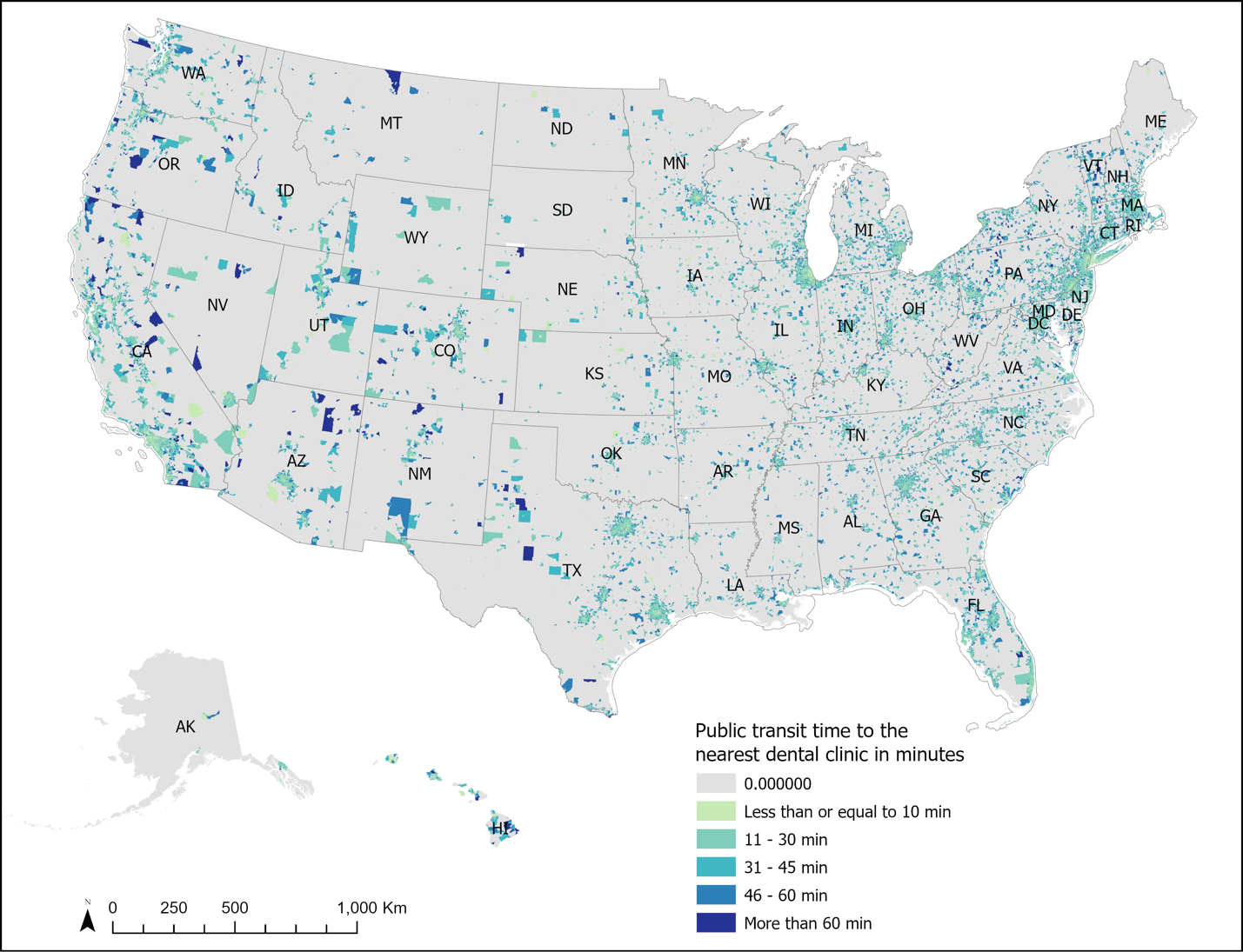


**Note.** Public transit time calculation to adult dental clinics. Authors’ analysis of data from 1) Dentist database from IQVIA; 2) American Community Survey 2022 estimates of population and the TIGER /line shapefiles from US Census Bureau. Categories are based on public transit time in minutes from the block group population center to the nearest dental clinics. Gray areas indicate block groups from where there was no available public transit route at the time of the Google Distance Matrix API request.

**Figure S4.** The difference in mean drive time between block groups with and without public transit.


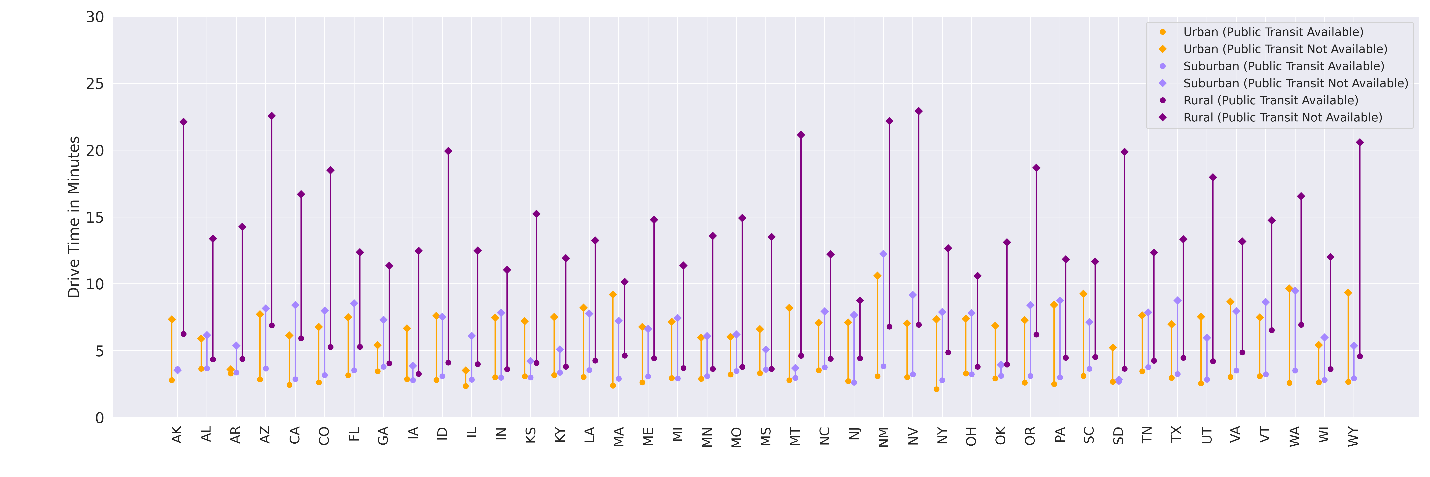


**Note.** Drive time calculation to adult dental clinics. Authors’ analysis of data from 1) Dentists database from IQVIA; Population without access to public transit refers to the population in block groups where public transit route was unavailable at the time of the Google Distance Matrix API request.

**Figure S5.** Cumulative population distribution by public transit time across census divisions


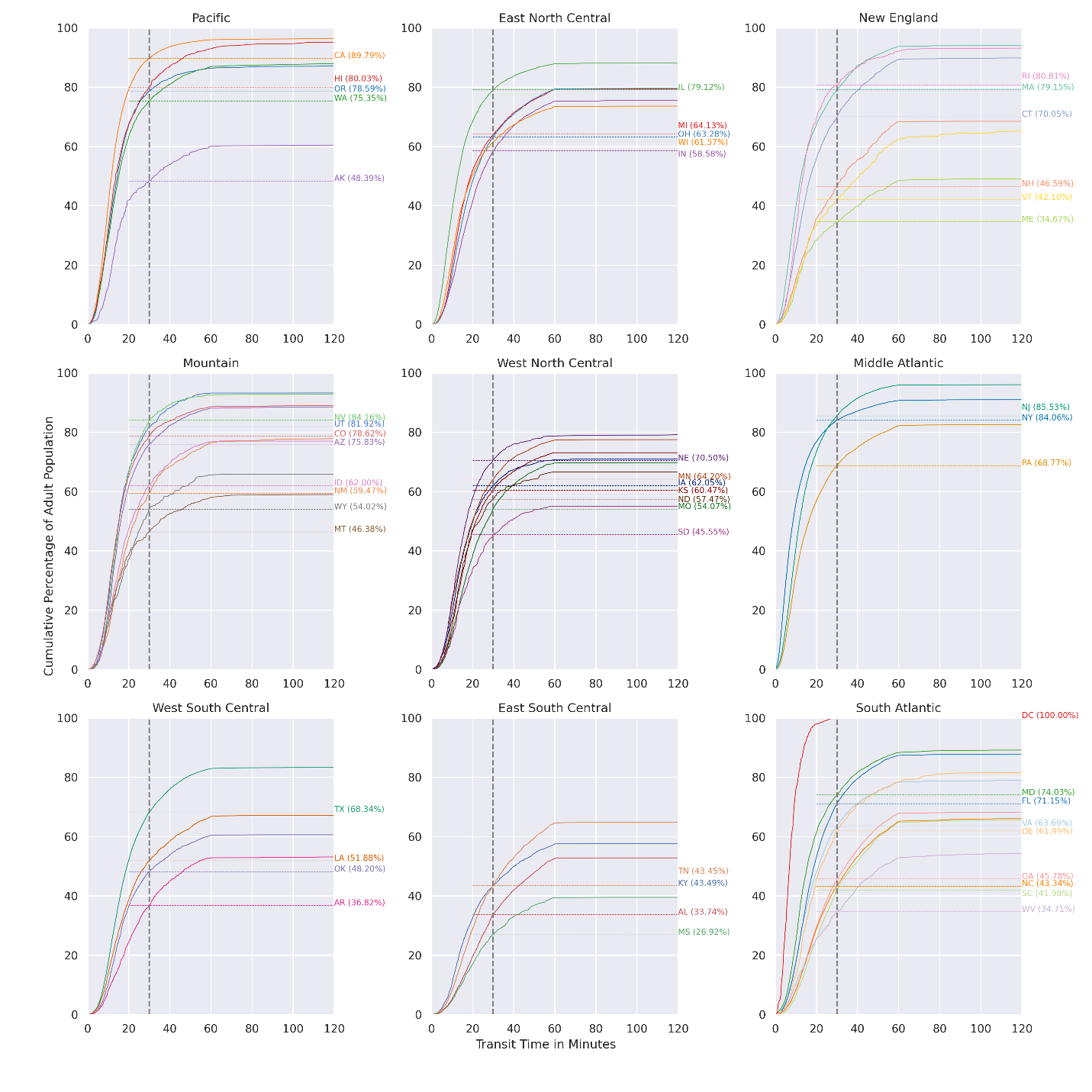


**Note.** Public transit time calculation to adult dental clinics. Authors’ analysis of data from 1) Dentists database from IQVIA; 2) American Community Survey 2022 estimates of the population from US Census Bureau. States are categorized according to the nine census divisions of the US. Calculation of cumulative population excluded these block groups where public transit is unavailable according to the Google API query

**Figure S6.** Cumulative population distribution by drive time across census divisions

**
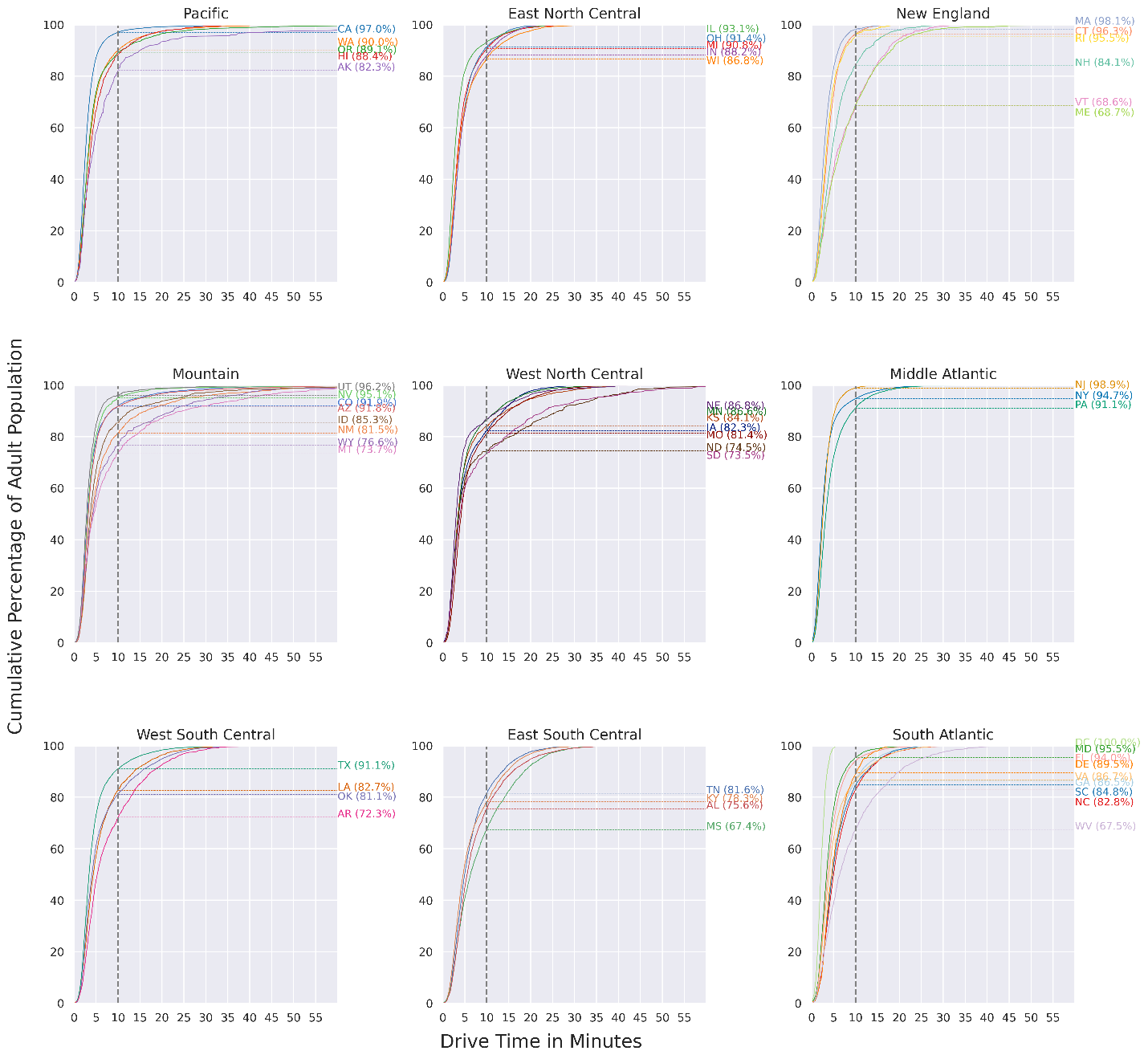
Note.** Drive time calculation to adult dental clinics. Authors’ analysis of data from 1) Dentists database from IQVIA; 2) American Community Survey 2022 estimates of the population from US Census Bureau. States are categorized according to the nine census divisions of the US.

**Table S1.** Descriptive statistics of drive time by census division in the US

| **Census Division** | **Urbanicity** | **Number of Block Groups** | **Mean drive time** | **Median drive time** | **Standard deviation** | **Minimum drive time** | **Maximum drive time** |
| --- | --- | --- | --- | --- | --- | --- | --- |
| East North Central | Urban | 11058 | 2.8 | 2.6 | 1.4 | 0.0 | 14.0 |
| East North Central | Suburban | 15437 | 3.2 | 2.8 | 1.8 | 0.0 | 28.8 |
| East North Central | Rural | 11168 | 8.9 | 7.8 | 5.8 | 0.0 | 86.1 |
| East South Central | Urban | 3726 | 3.5 | 3.2 | 1.7 | 0.0 | 11.5 |
| East South Central | Suburban | 3459 | 4.1 | 3.6 | 2.3 | 0.1 | 32.8 |
| East South Central | Rural | 7297 | 11.0 | 9.8 | 6.8 | 0.1 | 60.5 |
| Middle Atlantic | Urban | 10965 | 2.2 | 2.0 | 1.3 | 0.0 | 13.5 |
| Middle Atlantic | Suburban | 15727 | 2.9 | 2.6 | 1.8 | 0.0 | 27.0 |
| Middle Atlantic | Rural | 5892 | 9.0 | 7.6 | 6.4 | 0.0 | 83.9 |
| Mountain | Urban | 6629 | 2.8 | 2.6 | 1.4 | 0.1 | 16.9 |
| Mountain | Suburban | 6020 | 3.5 | 3.0 | 2.4 | 0.0 | 42.9 |
| Mountain | Rural | 4413 | 14.5 | 8.6 | 16.7 | 0.0 | 196.3 |
| New England | Urban | 2413 | 2.6 | 2.4 | 1.5 | 0.0 | 12.9 |
| New England | Suburban | 6026 | 3.2 | 2.9 | 1.9 | 0.0 | 19.2 |
| New England | Rural | 2890 | 9.3 | 7.4 | 7.9 | 0.0 | 106.7 |
| Pacific | Urban | 15088 | 2.5 | 2.3 | 1.4 | 0.0 | 23.8 |
| Pacific | Suburban | 14991 | 3.1 | 2.7 | 2.1 | 0.0 | 45.5 |
| Pacific | Rural | 5278 | 11.3 | 7.8 | 12.2 | 0.0 | 203.6 |
| South Atlantic | Urban | 11589 | 3.2 | 2.9 | 1.7 | 0.0 | 13.9 |
| South Atlantic | Suburban | 19616 | 3.9 | 3.5 | 2.2 | 0.0 | 28.0 |
| South Atlantic | Rural | 12978 | 10.0 | 8.7 | 6.4 | 0.0 | 133.7 |
| West North Central | Urban | 4986 | 3.0 | 2.8 | 1.4 | 0.0 | 15.2 |
| West North Central | Suburban | 5496 | 3.3 | 3.0 | 1.8 | 0.0 | 19.1 |
| West North Central | Rural | 7390 | 11.5 | 9.4 | 9.3 | 0.0 | 88.7 |
| West South Central | Urban | 11106 | 3.0 | 2.8 | 1.5 | 0.0 | 14.9 |
| West South Central | Suburban | 8480 | 3.7 | 3.2 | 2.9 | 0.0 | 83.6 |
| West South Central | Rural | 8988 | 10.8 | 8.8 | 8.1 | 0.0 | 91.0 |

**Note.** Descriptive statistics of drive time to adult dental clinics at nine census divisions of the US. Authors’ analysis of data from 1) Dentists database from IQVIA; 2) Block group counts from U.S. Census Bureau’s topologically Integrated Geographic Encoding and Referencing (TIGER/ Line Shapefiles) dataset using geographic identifiers (GEOIDs); 3) Urbanicity of Block groups was assigned using the National Center for Education Statistics (NCES) locale classification. Authors combined small towns and suburban into a single suburban category.

**Table S2.** Descriptive statistics of public transit time by census division in the US

| **Census Division** | **Urbanicity** | **Number of Block Groups** | **Mean drive time** | **Median drive time** | **Standard deviation** | **Minimum drive time** | **Maximum drive time** | |
| --- | --- | --- | --- | --- | --- | --- | --- | --- |
| East North Central | Urban | 10963 | 12.8 | 11.2 | 8.1 | 0.0 | 112.6 |  |
| East North Central | Suburban | 14802 | 18.2 | 14.9 | 12.3 | 0.1 | 256.6 |  |
| East North Central | Rural | 3752 | 30.9 | 28.9 | 21.0 | 0.1 | 774.7 |  |
| East South Central | Urban | 3599 | 19.0 | 16.4 | 11.3 | 0.1 | 140.8 |  |
| East South Central | Suburban | 2744 | 24.5 | 21.9 | 13.9 | 0.2 | 85.3 |  |
| East South Central | Rural | 1468 | 34.5 | 34.6 | 17.9 | 0.8 | 374.8 |  |
| Middle Atlantic | Urban | 10955 | 7.1 | 5.9 | 8.0 | 0.0 | 632.4 |  |
| Middle Atlantic | Suburban | 15344 | 15.6 | 12.4 | 12.3 | 0.0 | 553.3 |  |
| Middle Atlantic | Rural | 2391 | 35.7 | 34.1 | 27.3 | 0.1 | 777.2 |  |
| Mountain | Urban | 6600 | 14.2 | 12.4 | 8.2 | 0.1 | 87.8 |  |
| Mountain | Suburban | 5767 | 18.8 | 15.7 | 11.9 | 0.2 | 169.1 |  |
| Mountain | Rural | 1823 | 35.2 | 29.5 | 56.8 | 0.6 | 1472.9 |  |
| New England | Urban | 2408 | 10.6 | 8.7 | 8.1 | 0.0 | 92.7 |  |
| New England | Suburban | 5815 | 17.7 | 13.7 | 13.9 | 0.1 | 204.9 |  |
| New England | Rural | 1300 | 35.5 | 34.6 | 19.7 | 0.3 | 285.5 |  |
| Pacific | Urban | 15075 | 11.1 | 9.7 | 7.4 | 0.0 | 142.1 |  |
| Pacific | Suburban | 14782 | 15.2 | 12.5 | 11.4 | 0.1 | 318.7 |  |
| Pacific | Rural | 2882 | 35.4 | 29.3 | 43.2 | 0.6 | 1377.7 |  |
| South Atlantic | Urban | 11420 | 16.0 | 13.4 | 11.0 | 0.0 | 272.4 |  |
| South Atlantic | Suburban | 17987 | 21.9 | 18.2 | 17.1 | 0.1 | 883.1 |  |
| South Atlantic | Rural | 3968 | 39.4 | 36.1 | 48.1 | 0.1 | 968.7 |  |
| West North Central | Urban | 4958 | 14.6 | 12.5 | 8.9 | 0.2 | 130.7 |  |
| West North Central | Suburban | 5105 | 19.0 | 16.5 | 11.5 | 0.1 | 197.5 |  |
| West North Central | Rural | 2165 | 27.2 | 23.0 | 38.6 | 0.0 | 1262.7 |  |
| West South Central | Urban | 10983 | 15.7 | 13.3 | 10.2 | 0.1 | 245.9 |  |
| West South Central | Rural | 7446 | 21.0 | 17.8 | 12.9 | 0.4 | 238.5 |  |
| West South Central | Suburban | 2566 | 33.6 | 30.4 | 36.2 | 0.9 | 1381.4 |  |

**Note.** Descriptive statistics of public transit time to adult dental clinics at nine census divisions of the US. Authors’ analysis of data from 1) Dentists database from IQVIA; 2) Block group counts from U.S. Census Bureau’s topologically Integrated Geographic Encoding and Referencing (TIGER/ Line Shapefiles) dataset using geographic identifiers (GEOIDs); 3) Urbanicity of Block groups was assigned using the National Center for Education Statistics (NCES) locale classification. Authors’ combined small towns and suburban into a single suburban category.

**Table S3.** Model fit indices for the spatial lag regression models for drive time

| **Model** | **AIC** | **BIC** | **logLik** | **deviance** | **df_resid** | **McFadden_R2** |
| --- | --- | --- | --- | --- | --- | --- |
| **Drive time** | 24779.2 | 24934.8 | -12374.6 | 24749.2 | 236626.0 | 0.88 |
| **Stratified drive time for suburban** | 2887.2 | 3010.1 | -1430.6 | 2861.2 | 94479.0 | 0.93 |
| **Stratified drive time for rural** | 21413.5 | 21531.7 | -10693.8 | 21387.5 | 65820.0 | 0.77 |

**Note.** Model fit indices of spatial lag regression of drive time to the nearest dental clinics. Author estimates 1) Akaike Information Criterion (AIC); 2) Bayesian Information Criterion (BIC); 3) Log-Likelihood (LogLik); 4) Residual Degree of Freedom (df_resid); and 4) McFadden’s R^2^ (Pseudo R^2^)

**Table S4.** Model fit indices for the spatial lag regression models for public transit

| **Model** | **AIC** | **BIC** | **logLik** | **deviance** | **df_resid** | **McFadden_R2** |
| --- | --- | --- | --- | --- | --- | --- |
| **Public transit time** | 27305.6 | 27457.6 | -13637.8 | 27275.6 | 186932.0 | 0.84 |
| **Stratified public transit time for urban** | 3490.6 | 3610.7 | -1732.3 | 3464.6 | 75728.0 | 0.86 |
| **Stratified public transit time for suburban** | 12976.2 | 13098.4 | -6475.1 | 12950.2 | 89068.0 | 0.86 |
| **Stratified public transit time for rural** | 9491.7 | 9595.8 | -4732.9 | 9465.7 | 22112.0 | 0.69 |

**Note.** Model fit indices of spatial lag regression of public transit time to the nearest dental clinics. Author estimates 1) Akaike Information Criterion (AIC); 2) Bayesian Information Criterion (BIC); 3) Log-Likelihood (LogLik); 4) Residual Degree of Freedom (df_resid); and 4) McFadden’s R2 (Pseudo R2)

**Table S5.** Characteristics of hotspots for driving and public transit times

|  | **Drive Time Hotspots**  (n= 38,509) | **Public Transit Time Hotspots**  n=32,333 |
| --- | --- | --- |
|  | **Odd Ratio [95% CI]** | |
| **Neighborhood Types** |  |  |
| Urban Area | Reference | Reference |
| Suburban Areas | 5.0 [3.3, 7.7] **** | 1.4 [1.3, 1.6] **** |
| Rural Areas | 6.5 [4.2, 10.0] **** | 2.4 [2.2, 2.7] **** |
| **Adult Population Density** | 0.1 [0.0, 0.3] **** | 0.1 [0.0, 0.1] **** |
| **Uninsured adult population (%)** | 1.0 [0.9, 1.0] | 1.0 [1.0, 1.1] |
| **Black segregation** |  |  |
| Dissimilarity index (1^st^ to 3^rd^ quartile) | Reference | Reference |
| Dissimilarity index (4^th^ quartile) | 1.2 [1.1, 1.3] **** | 1.0 [0.9, 1.1] |
| **Hispanic segregation** |  |  |
| Dissimilarity index (1^st^  to 3^rd^ quartile) | Reference | Reference |
| Dissimilarity index (4^th^ quartile) | 1.1 [1.0, 1.2] ** | 1.2 [1.0, 1.3] *** |
| **Social Deprivation Index (SDI)** |  |  |
| SDI-1 (least deprived) | Reference | Reference |
| SDI-2 | 1.3 [1.2, 1.5] **** | 2.1 [1.9, 2.3] **** |
| SDI-3 (most deprived) | 1.7 [1.5, 2.0] **** | 3.7 [3.2, 4.3] **** |

**Note.** Travel time calculation to the adult dental clinics. Authors’ analysis of data from 1) Dentists database from IQVIA; 2) American Community Survey 2022 estimates of population. 3) AIC, BIC, and log-likelihood for drive time model are 57796, 57931, and –28885. 4) AIC, BIC, and log-likelihood for public transit time model are 65584, 65716, and –32779. *p < 0.10, **p < 0.05, ***p < 0.01 ****p < 0.001.

**Table S6.** Lag odd for the spatial lag regression models for driving and public transit times

|  | **Drive Time Hotspots**  (OR, 95% CI) | **Public Transit Time Hotspots**  (OR, 95% CI) |
| --- | --- | --- |
| **Lag hotspot** | 23753.8 [20375.7, 27691.9] **** | 10766.8 [9526.5, 12168.5] **** |
| **Adult Population Density** | 0.10 [0.0, 0.2] **** | 1.11 [0.8, 1.5] |
| **Lag Uninsured adult population** | 1.10 [1.0, 1.2] ** | 1.50 [(1.4, 1.6] **** |
| **Lag Black Dissimilarity** | 1.10 [0.9, 1.2] | 0.59 [0.5, 0.7] **** |
| **Lag Hispanic Dissimilarity** | 0.90 [0.7, 1.0] ** | 0.93 [0.8, 1.1] |
| **Lag Social Deprivation Index** | 0.80 [0.7, 0.9] **** | 0.27 [0.3, 0.3] **** |

**Note.** Travel time calculation to the adult dental clinics. Authors’ analysis of data from 1) Dentists database from IQVIA; 2) American Community Survey 2022 estimates of population; 3) lag odd ratios (OR) were estimated from spatial lag logistic; 4) AIC, BIC, and log-likelihood for drive time model are 57796, 57931, and –28885; 5) AIC, BIC, and log-likelihood for public transit time model are 65584, 65716, and –32779. *p < 0.10, **p < 0.05, ***p < 0.01 ****p < 0.001.

**REFERENCES**

Chambers, B.D., Erausquin, J.T., Tanner, A.E., Nichols, T.R., Brown-Jeffy, S., 2018. Testing the Association Between Traditional and Novel Indicators of County-Level Structural Racism and Birth Outcomes among Black and White Women. J. Racial and Ethnic Health Disparities 5, 966–977. https://doi.org/10.1007/s40615-017-0444-z

ESRI, 2012. World Geocoding [WWW Document]. URL https://hub.arcgis.com/content/305f2e55e67f4389bef269669fc2e284/about (accessed 7.5.24).

ESRI, n.d. Generate Origin Destination Cost Matrix (Ready To Use)—ArcGIS Pro | Documentation [WWW Document]. URL https://pro.arcgis.com/en/pro-app/latest/tool-reference/ready-to-use/itemdesc-generateorigindestinationcostmatrix.htm (accessed 1.22.25).

Google, 2024. Distance Matrix API overview.

IQIVIA, 2023. ONEKEY Fact Sheet.

US Census Bureau, 2022a. ACS Demographic and Housing Estimates, five-year estimate 2022– All block groups US.

US Census Bureau, 2022b. DEC Demographic and Housing Characteristics, Decennial Census 2020 – All blocks US.
